# Cancer care during the Covid-19 pandemic from the perspective of patients and their relatives: A qualitative study

**DOI:** 10.1016/j.heliyon.2023.e19752

**Published:** 2023-09-14

**Authors:** Andrea J. van Puffelen, Lisa J. van der Sar, Frederique Moerman, Manuela Eicher, Wendy H. Oldenmenger

**Affiliations:** a Erasmus MC Cancer Institute, University Medical Center Rotterdam, Department of Medical Oncology, P.O. Box 2040, 3000 CA Rotterdam, the Netherlands; b Institute of Higher Education and Research in Healthcare (IUFRS), Faculty of Biology and Medicine, University of Lausanne, Lausanne, Switzerland; c Lausanne University Hospital (CHUV) Department of Oncology, Lausanne, Switzerland

**Keywords:** Covid-19, Oncology, Patient experiences, Family caregivers, Cancer nurses

## Abstract

**Objective:**

The Covid −19 pandemic has had a major influence on the organization of cancer care. Little is known about how patients with cancer and their relatives experienced this period. This study explored these experiences and levels of distress and resilience of Dutch cancer patients and their family caregivers during the Covid-19 pandemic.

**Methods:**

The qualitative design included in-depth interviews with cancer patients and their family caregivers to explore their experiences. The distress thermometer (NCCN-DT) and resilience questionnaire (CD-RISC2) were used for contextualizing. Data were analyzed by thematic analysis and descriptive statistics.

**Results:**

40 patients with breast cancer, lung cancer, colorectal cancer, or melanoma who received active systemic anti-cancer therapy, were included with a median age of 60 years[SD11.1]. We also included fourteen family caregivers with a median age of 60 years [SD8.6].

Five themes were identified: (1) Living with cancer during Covid-19, (2) Changes in cancer care, (3) Information and support, (4) Safety inside the hospital, and (5) Impact of vaccination. The mean score of NCCN-DT was 2.9[SD2.4] for patients and 4.3[SD2.7] for family caregivers. Mean score of CD-RISC2 was 6.6[SD1.4] for patients and 7.2[SD1] for family caregivers.

**Conclusions:**

Patients felt vulnerable during the pandemic and were strict in following the safety precautions. The limited companionship of family caregivers was experienced as the biggest restraint. In general, they felt safe inside the hospital. Vaccination brought some relief. Patients were satisfied with the provided support, but areas were identified which are amenable for redesigning care processes.

## Highlights

• Covid-19 added an extra risk upon the experienced vulnerability of cancer patients
• Patients worried most about the continuity of their treatment during the pandemic
• Limited companionship was a negative experience for patients and family caregivers
• The pandemic also brings opportunities for care redesign

## 1 Introduction

The coronavirus disease 2019 (Covid-19) pandemic had since its start an impact on almost every aspect of society. During several waves of the pandemic, healthcare was put under pressure due to the high influx of Covid-19 patients, the consequences for the regular care, and the high rates of sick leave and mental health issues among healthcare workers [1].This limited the capacity of the health care systems in general, wherefore many changes were made in the delivery of care.

Patients with comorbid conditions seemed to be more at risk of manifesting complications and suffering severe consequences of a Covid-19 infection; this also applies to patients with cancer due to their immunocompromised status as a result of the nature of cancer and/or the systemic anti-cancer treatments (SATC) [[2], [3], [4]].

The uncertainties about the increased risk of worse outcomes of a Covid-19 infection for cancer patients, affected the organization of cancer care in particular [5,6]. Access to health care is an important factor, that might affect treatment and care outcomes for patients with cancer, due to their need for life-sustaining treatment. Delay in treatment has a negative effect on the state of the disease. This major concern competed with the concern about an increased risk of a Covid-19 infection; it puts the patient in a position to choose between their treatment wherefore a hospital visit is required, and minimizing the risk of getting infected by staying home [7].

Patients with cancer and their family caregivers already have a higher risk of depression and anxiety, due to the disease and therapy [8]. Psychological distress can have a negative impact on the quality of life, the response to treatment, performance status, medical care satisfaction, and survival [9]. An increased risk of severe infection and associated mortality risk, changes in cancer care, and the social isolation from stay-at-home restrictions may have added new sources of stress and anxiety, along with concerns about a worsened cancer prognosis [10,11]. This is confirmed in earlier studies, performed in different countries, e.g. Israel, Switzerland and Denmark [[12], [13], [14]]. Loneliness, and social isolation itself, besides emotional distress, can contribute to negative clinical outcomes for patients with cancer [15].

At the start of this study, most of what we knew about the experiences of patients with cancer and their relatives with care during the Covid-19 pandemic has been analyzed based on quantitative research. This study adds an in-depth sight into this information, due to the qualitative study design. The primary aim of this study was to explore the experiences of Dutch cancer patients and their family caregivers in general and with cancer care, during the two first waves of Covid-19 until the beginning of the vaccination campaign in the Netherlands. The secondary aim was to contextualize the results with the levels of distress and resilience.

## 2 Methods

### 2.1 Study design

In this qualitative study, we used an explorative approach based on thematic analysis without a predefined endpoint. We conducted semi-structured in-depth interviews with cancer patients under active SACT and their family caregivers. We also measured the levels of distress and resilience, to contextualize the psychological impact of Covid-19. This study is conducted in several European countries, based on the same protocol, but adapted to local circumstances. The COREQ guideline is used for reporting (supplementary 1).

### 2.2 Participants

The enrollment took place between November 2020 and July 2021 at the outpatient clinic of the Erasmus University Medical Center, Rotterdam, the Netherlands. The outpatient clinic is mainly visited by patients with SACT only. Therefore, patients with combination therapy (with surgery and/or radiotherapy) are excluded.

Patients were eligible when they were adults (≥18 years) with a confirmed diagnosis of lung cancer, colorectal cancer, breast cancer or melanoma, who received active SACT with curative or palliative intent during the Covid-19 pandemic, and were Dutch speaking. Exclusion criteria were a life expectancy of less than three months, combination therapy with surgery and/or radiotherapy, hospitalization, a positive Covid-19 test in the past, and inability to give informed consent. Family caregivers were included when they were appointed as a family caregiver by the patient, adults, Dutch-speaking, and had given informed consent.

### 2.3 Sample size

Due to the exploratory nature of this study, the sample size could not be determined a priori. Commonly, sample sizes for in-depth interviews range from 12 to 20 participants for maximum variation sampling in qualitative studies and up to 40 participants in population-based patient experiences studies [16].For recruiting different subgroups, the strategy of purposive sampling was used. We included 54 participants, of which 40 patients and 14 family caregivers. This sample size was sufficient for achieving a state of data analysis where no new codes, subthemes, and themes could be identified.

### 2.4 Study procedures

The researchers (AP/LS/FM) screened the scheduled admissions at the daycare unit for potential candidates and approached the patients during admission. Patients were verbally informed about the study and written information was provided. Patients were asked to identify family caregivers and asked for permission to approach them. After written informed consent, the participants received a paper-and pencil questionnaire including distress and resilience measures. Depending on the participants’ wishes, the interview (by AP/LS/FM) took place through a video- or phone consultation at a time and place chosen by the participant. The interviews lasted an average of approximately 30 min and were all audio recorded and transcribed verbatim.

### 2.5 Data gathering and measurements

An interview guide developed by the research team (AP/LS/FM/WO) based on a previous study under the same protocol [14] and experiences from healthcare workers, was used to ensure consistency among interviewers and interviews (supplementary 2). Patients were asked about their experiences during de pandemic in general and with health care and how it affected their daily life. The guide is pilot tested and finalized with minor adjustments to improve the reflection of patient experiences.

Before the interview, a brief questionnaire was carried out for measuring their experienced distress and resilience. Level of distress was measured with the Dutch version of de National Comprehensive Cancer Network Distress Thermometer (NCCN-DT), which is adequately validated in multiple studies, for patients as well for family caregivers [17,18]. The NCCN-DT is a Likert scale from 0 (no distress) to 10 (severe distress), with an established cut-off score of 4 for further screening. Higher scores indicate a higher level of distress [8,19].

Resilience was measured with the Dutch version of the 2-item Connor-Davidson Resilience Scale (CD-RISC2) including the items ‘able to adapt to change’ and ‘tend to bounce back after illness or hardship’. Both items have 5-point response options from 0 (not at all true) to 4 (true nearly all the time), with higher scores reflecting higher resilience [[20], [21]]. During the interviews, patients were invited to explain their scores for both questionnaires.

Characteristics about the participants and their disease were collected from their medical records (e.g. age, gender, diagnosis and stage, current treatment, and marital status). Results were discussed with stakeholders (e.g. health care professionals).

### 2.6 Data analysis

For thematic analyzing, the verbatim transcriptions were entered into Nvivo software (version 12, QRS International). Thematic analysis is a method for identifying, analyzing, and reporting patterns within data. In this study, the steps outlined by Braun & Clarke [16] were followed. These consist of (1) familiarizing with the data, (2) generating initial codes, (3) searching for themes, (4) reviewing themes, (5) defining and naming themes, and (6) producing the report.

Data analysis was conducted concurrently with data collection to assess for data saturation and for building up the coding scheme. Descriptive analysis (mean and standard deviation) of the characteristics of the participants, their disease, and the levels of distress and resilience, were conducted using SPPS version 27. The scores of the two questions from CD-RISC2 were combined in this analysis, by summing up the separate scores and taking the average score.

### 2.7 Ethical considerations

The study was approved by the Medical Ethics Review Committee of the Erasmus MC (no. 2020-0598). All participants received verbal and written information and provided verbal and written consent before the start of the interview. Participation was voluntary and participants were informed that they were allowed to withdraw their consent at any time. The transcripts and data were pseudonymized and stored on a secured server at the research center.

## 3 Results

### 3.1 Demographic and clinical characteristics

In total, 40 patients (19 males), with lung cancer (11), breast cancer (9), colorectal cancer (7), and melanoma (9) were included as well as 14 family caregivers of whom 7 males. The mean age of the respondents was 60 years (SD 10.6, range 33–89). Most patients had metastatic disease (92%) and they were all receiving active treatment (Table 1).

**Table 1:** Characteristics of participants.

	Patients N = 40 N (%)	Family caregivers N = 14 N (%)
Age, years Mean [SD]	60.1 [11.1]	59.8 [8.6]
Gender		
**Male**	19 (47)	7 (50)
**Female**	21 (53)	7 (50)
Marital status		
**Married**	28 (70)	13 (92.9)
**Single**	3 (7.5)	0
**Divorced**	7 (17.5)	0
**Civil Partnership**	2 (5)	0
**Missing**	0	1
Type of cancer		n/a
**Lung cancer**	13 (32.5)	
**Breast cancer**	10 (25)	
**Colorectal cancer**	7 (17.5)	
**Melanoma**	10 (25)	
Disease setting		n/a
**Curable disease**	3 (7.5)	
**Incurable disease**	37 (92.5)	
Type of treatment		n/a
**Chemotherapy**	11 (27.5)	
**Immunotherapy**	21 (52.5)	
**Chemo- & Immunotherapy**	3 (7.5)	
**Immuno- & Hormone therapy**	1 (2.5)	
Missing	4	
Nurse Consultation		n/a
**Yes**	31 (77.5)	
**No**	9 (22.5)	
Distress Thermometer Mean [SD]	2.9 [2.4]	4.3 [2.7]
CD-RISC2 (combined) Mean [SD]	6.6 [1.4]	7.2 [1]

### 3.2 Themes

**Fig. 1:**
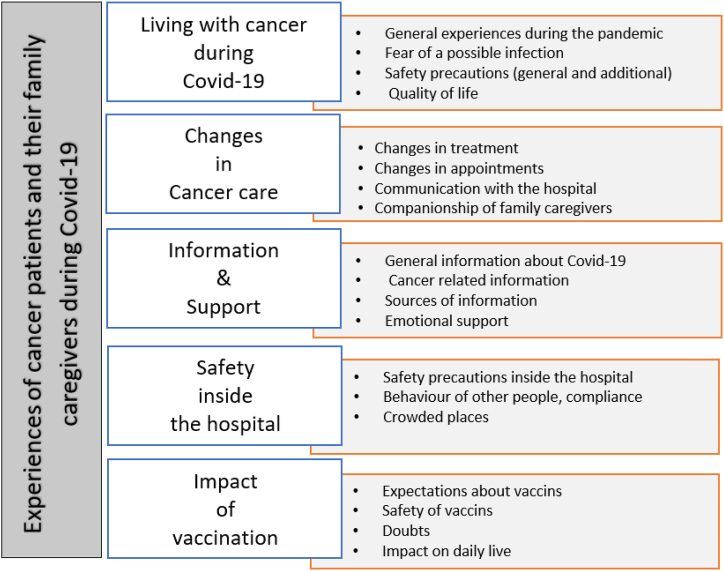
Experiences of patients with cancer and their family caregivers during Covid-19.

From the interviews, five themes evolved: (1) Living with cancer during Covid-19, (2) Changes in cancer care, (3) Information and support, (4) Safety inside the hospital, and (5) Impact of vaccination (Fig. 1).

### 3.3 Distress & resilience

The mean score of patients of the NCCN-DT was 2.9 on a 0–10 scale, with differences between patients with lung cancer (M4.8) and patients with melanoma (M1.6). Patients with colon carcinoma and breast cancer scored respectively a 2.5 and a 2.3. For resilience, patients scored a 6.6 on a 0–8 scale. Patients with melanoma scored the highest (M7.4), associated with a higher resilience and patients with colon carcinoma scored the lowest (M6).

Caregivers scored a 4.3 for distress and a 7.2 for resilience. When asked for an explanation for the personal scores on NCCN-DT and CDRISC2, patients reflected on their experience describing they had found a way to accept the situation; they had learned to accept things since they were diagnosed with cancer.

“At the moment, I’m not really worried. I think I have accepted the situation and making the best out of it, that’s also what cancer has taught me (R010: patient with colon carcinoma, male, 69)”.

### 3.4 Living with cancer during Covid-19

During the pandemic, patients described themselves as very careful in applying safety measures, due to their experienced vulnerability. It was unclear to them what the impact of an infection could be. Some patients were told that it was likely that they could get very ill and even end up in the Intensive Care Unit or, in the worst case, could die. This uncertainty caused a lot of stress and led patients to take additional restrictions on top of the national safety precautions. Many of them already used facemasks in the public area, before this was mandatory. They were also very strict in receiving home visits and holding distance from other people. Sometimes this felt very lonely. When asked what they missed the most, many patients answered that they missed physical contact with their closest family and friends, like a hug or a handshake. Some patients described the difficulty of knowing about a limited life expectancy and not being able to spend it the way one wanted, due to the pandemic.

“You know, I have metastatic disease. I wanted to spend this time making memories with my family, which was almost impossible. Yes, I found that very difficult (R004: patient with breast cancer, female, 60)”.

Some patients noted that they were already used to avoid (physical) contact, because of their immune-compromised state. It was experienced as a relief that this became the normal situation for everyone. Though patients saw Covid-19 as a threat to their health, most of them felt that cancer was their main concern.

“It won’t happen to me that I die of Covid. I have cancer and that’s what will cause my death, at the end (R005: patient with breast cancer, female, 52)”.

Patients worried about the continuity of their treatment, especially in the first weeks of the pandemic. They were relieved to find out that their treatment was not at risk and could continue as before.

Family caregivers described being very careful at work, in shops, and in public spaces to avoid any infection. They expressed worries about the vulnerability of their loved ones and describes how they supported the patients with self-initiated precautions and restrictions.

“Of course, I’m more worried about her, because of her vulnerability. When I get infected, there is a big chance that I will infect her too, we are living together. Yes, I’m really paying more attention to that, than I would when she was not ill (RA004: family caregiver, male, 62)”.

### 3.5 Changes in cancer care

Patients who received immunotherapy reported that, in the first weeks, changes were made in their treatment schedule. They trusted their physician that this would not have a negative effect on their cancer. In fact, some of them were glad about this, because it saved them visits to the hospital.

Also, patients, independent of the type of treatment, indicated that they had more consults by phone, instead of consults at the outpatient clinic. Most of them did not experience this as negative, describing that it saved them a trip to the hospital. They noted that the suitability of telephone consults (TC) depends on the type of visit. Most patients preferred to see their physician in person to discuss the results from scans for evaluating the state of the disease. For most patients, this was organized this way. In most cases, family caregivers described that they were not listed in during the TCs. Some patients experienced the TCs as negative because they lacked eye contact and body language. The use of web consults was available, but no patient had experience with this type of consultation. Though, almost all patients could imagine that this could be a beneficiary option in the future and may even be preferable above TCs.

The limited companionship of family caregivers during the hospital visit was experienced as difficult; they could accompany their relatives during the consults at the outpatient clinic, but not at the daycare center. Since most of these visits were combined, patients described they went alone to the hospital for all the appointments or relatives had to wait someplace else during the admission at the daycare center.

“At some points at the daycare unit, I really felt miserable, like when it was difficult to insert an IV or when I had to wait for a long period. Then, I really missed my partner, especially because I knew he was inside the same building, but he could not be with me (R019: patient with melanoma, female, 58)”.

The relatives felt not comfortable with leaving the patient behind. A part of the patients felt lonely during the admission, but most of them accepted the situation. Some patients found it even a relief, not feeling responsible for their relatives. As a positive change during the pandemic patients described the apparent shorter waiting time at different locations inside the hospital.

### 3.6 Information & support

Patients felt informed about the pandemic, in general by the media. Some of them consulted patient associations and read scientific papers. Patients found it difficult to gain insight into the possible consequences for their personal situation due to their disease. Therefore, they relied on their physician for receiving relevant information.

Most patients experienced adequate support from their health care professionals (HCP) and did not experience any change, compared with the situation before the pandemic.

“When you need help, they were always there. Even when they were very busy, and they were, they took their time for you(R038: patient with breast cancer, female, 68)”.

A part of the patients reported a lack of social support from their physician; these paid a lot of attention to the possible medical consequences of Covid-19, but not to the emotional ones. Though, patients who had a nurse consultant, were very positive about their received emotional support. They could easily contact their nurse consultant with all sort of questions; for some this contact was even better than before. Patients had also positive experiences with the support from the nurses at the daycare center.

Relatives indicated that they had not received any support, but they did not expect this either. Though, they were all satisfied with the support for their loved ones. Some of them had organized support for themselves, mostly outside the hospital.

### 3.7 Safety inside the hospital

In general, patients and their relatives were satisfied with the efforts made in the hospital for preventing a possible Covid-19 infection. They were very positive about the support from the hosts at the central hall. This made them feel safe during a visit.

Some places were still crowded, like the central hall and the waiting areas. Also, some patients were irritated by the behavior of other people and felt there was not enough control over compliance with safety measures. Many patients noted they had some contradictory feelings about visiting the hospital: they wanted to visit for their treatment, but on the other hand, they liked to avoid human contact as much as possible.

“The hospital is the only place where I see other people, it is a little bit conflicting for me. At home, I try to avoid contact with other people, but here, there are people everywhere. I do understand, but it feels uncomfortable (R017: patient with colon carcinoma, male, 63)”.

### 3.8 Impact of vaccination

For most patients, the possibility of vaccination brought relief in a time of fear. They felt less vulnerable, although they had concerns about the effectiveness of the vaccine for themselves, due to their immune-compromised state. Another major concern was, whether the vaccination itself or possible side effects, could affect their anticancer treatment.

“I fear that I will get ill from the vaccine and then, I cannot get my chemo (R033: patient with lung cancer, female, 59)”.

Though, vaccination had little impact on daily life; patients maintained strict in following the safety precautions, to prevent themselves from getting infected.

## 4 Discussion

In this thematic analysis, we explored the experiences of patients with cancer and their family during the Covid-19 pandemic. Patients felt already vulnerable due to their disease, and Covid-19 carried an additional risk. Cancer remained the biggest threat. They worried most about their vulnerability and possible negative effects on the continuity of their treatment. This is in accordance with earlier studies [5,[13], [14], [15],^22^]. Another concern of patients and family caregivers is that they could not live the life they want at this stage, due to the restrictions and the fear of an infection. It is important to pay attention to these topics since they may affect patients' and family caregivers’ well-being and quality of life, which indirectly could have an impact on treatment efficacy; cancer itself has a negative impact on the emotional well-being of patients and the effects of the pandemic could worsen this. On the other side, patients reported a low score of distress (M2.9) and a high score of resilience (M6.6), which is consistent with findings from other studies [[13], [14], [15],^23^]. The differences in distress between patients with lung cancer (M4.7) and melanoma (distress M1.6) might be explainable by the differences in life expectancy and different predictions of benefit of treatments. Though, the sample size of the subgroups are small, what makes it difficult to draw conclusions. However, this might be an interesting topic for further research. Of note, the reported concerns from qualitative interviews provided a much more detailed understanding of the distress and resilience of patients during the pandemic situation. It seems that patients had already adjusted their way of living due to their cancer which was considered as a resource during the pandemic, which is in line with the findings from the studies in several European countries under the same protocol [13,14]. As the study of Schellekens & Van der Lee [24] concluded, half of their included patients felt more at peace due to the lockdown, which gave them time to reflect on their lives. Altogether, this may explain the high levels of resilience.

The companionship of family caregivers was limited to the outpatient clinic and not allowed at the daycare unit. Our analysis shows how important it is for patients that they can bring someone with them to the hospital. As reported by patients, the support from relatives helped them process the received information, and undergo procedures, like taking blood samples or administration of medication. Fulfilling this role is important for family caregivers. They found it difficult to leave the patient alone in the hospital, not being able to support their loved ones as they wished. It is highly recommended to take the companionship of relatives into account in the organization of care, for the wellbeing of the patients and family caregivers as well.

An interesting point is the discrepancy in distress between patients and family caregivers. It seemed that the latter experienced a higher level of distress (M2.9 vs. M4.3). This is also shown in the study of van Roij et al. [9]. Patients experienced an increased emotional burden during the Covid-19 pandemic [5]. It is imaginable that this affected the burden of family caregivers since they were constantly balancing between the patient's needs and their own, as is shown in earlier studies [25,26]. Factors as caregiver psychological health, social isolation and limited family and social support are important contributors to high levels of caregiver burden [30]. These factors were even more pronounced during the pandemic, what could have led to an increased level of distress, together with their concerns about the vulnerability of the patient and the fear to infect them.

The changes in cancer care during the pandemic also bring opportunities for redesigning the healthcare system. Before the pandemic, all the visits took place physically at the outpatient clinic, independent of the type of visit. The results of this study show that most patients did not experience telephone consults worse than a face-to-face visit. It was an advantage that they did not have to travel for every appointment and they felt safer at home. This is also shown in other studies [27,28]. This suggests that the use of TC's could reduce the burden for patients. Patients had no experience with video consults, but they were positive about the interviews using video connection for this study. The use of video consults could also help with involving family caregivers, as is suggested [14]. This can have a positive impact on the experienced distress for this group. Also, some patients pointed out that they missed eye contact and body language during TC's. The use of video consults instead of TC's, could meet these needs. Using telephone consults and video consults, can contribute positively to the organization of care without affecting the quality. It must be noted that not every physical visit is suitable to be replaced with TC's or video consults. This should be taken into account before further implementation.

### 4.1 Strengths and limitations

One of the strengths is the large sample size of this qualitative study. This enabled us to reach data saturation. Another strength is the integration of quantitative and qualitative data, which may limit the influence of social desirability bias, a well-known limitation of qualitative research.

This study was performed in a single institution, which may influence the generalizability. However, the findings are generally in line with the results from a larger Dutch quantitative study [5,29]. Another limitation is that the period between the first and last interview was quite long (seven months). De pandemic evolved rapidly, which might have caused different circumstances for the individual respondents. Another limitation is that not all family caregivers could be interviewed in this study, however, data saturation was also received for this group.

## 5 Conclusion

Patients felt already vulnerable due to their disease, and Covid-19 had put an extra risk upon that. Most patients took extra precautions, but they were already used to be careful with their health, due to their disease. Vaccination brought relief but had minimal effect on daily life. Cancer remains the biggest threat, and patients were most worried about the continuity of their treatment. Small changes were made in cancer care, except for the replacement to TCs and the limited companionship of family caregivers. This was experienced as a burden for patients, as well as for their relatives. The Covid-19 pandemic has led to adjustments in cancer care, which offer opportunities in the regular setting for reducing the burden for patient and simplifying the organization of care. Telephone consults could be used more often, and video consults could be considered a good alternative for overcoming the negative aspects of TC's Family caregivers should not be overlooked for their own psychological wellbeing, and the wellbeing of patients as well.

## Funding

This study was supported by the Erasmus MC Evidence Based Care by Nurses (EBCN). The funder had no active role in the conduct, analysis or report of the study.

## Author contribution statement

Andrea J. van Puffelen: Performed the experiments; Analyzed and interpreted the data; Wrote the paper.

Lisa J. van der Sar; Frederique Moerman: Performed the experiments; Analyzed and interpreted the data.

Manuela Eicher: Conceived and designed the experiments.

Wendy H. Oldenmenger: Conceived and designed the experiments; Contributed reagents, materials, analysis tools or data.

## Data availability statement

Data will be made available on request.

## Declaration of competing interest

The authors declare that they have no known competing financial interests or personal relationships that could have appeared to influence the work reported in this paper.
