# Supplementary material for: Cancer care during the Covid-19 pandemic from the perspective of patients and their relatives: A qualitative study
Source: Heliyon. 2023 Sep 14;9(9):e19752. doi: 10.1016/j.heliyon.2023.e19752 (PMC10559054; doi:10.1016/j.heliyon.2023.e19752)
Supplement: Multimedia component 1 [file mmc1.docx]

**Supplementary 1: Consolidated criteria for reporting qualitative studies (COREQ): 32-item checklist**

**Cancer care during the Covid-19 pandemic from the perspective of patients and their relatives: a qualitative study**

Andrea van Puffelen¹; Lisa J. van der Sar¹; Frederique Moerman¹; Manuela Eicher² Wendy H. Oldenmenger¹

¹ Erasmus MC Cancer Institute, University Medical Center Rotterdam, Department of Medical Oncology, P.O. Box 2040, 3000 CA Rotterdam, the Netherlands

²Institute of Higher Education and Research in Healthcare (IUFRS), Faculty of Biology and Medicine, University of Lausanne, Lausanne, Switzerland / Lausanne University Hospital (CHUV) Department of Oncology, Lausanne, Switzerland.

| **No** | **Item** | **Guide questions/description** | **Reporting** |
| --- | --- | --- | --- |
| **Domain 1: Research team and reflexivity** |  |  |  |
| Personal Characteristics |  |  |  |
| 1. | Interviewer/facilitator | Which author/s conducted the interview or focus group? | Andrea van Puffelen  Lisa van der Sar  Frederique Moerman |
| 2. | Credentials | What were the researcher's credentials? *E.g. PhD, MD* | Andrea van Puffelen,RN MSc  Lisa J van der Sar, MSc  Frederique Moerman, MSc  Manuela Eicher, RN PhD  Wendy H. Oldenmenger, RN PhD |
| 3. | Occupation | What was their occupation at the time of the study? | Andrea van Puffelen: Nurse Practitioner at affiliation 1  Lisa J. van der Sar: medical master student at affiliation 1  Frederique Moerman: medical master student at affiliation 1  Manuela Eicher: Professor at affiliation 2  Wendy H. Oldenmenger: Assistant professor at affiliation 1 |
| 4. | Gender | Was the researcher male or female? | Andrea van Puffelen: Female  Lisa J. van der Sar: Female  Frederique Moerman: Female  Manuela Eicher: Female  Wendy H. Oldenmenger: Female |
| 5. | Experience and training | What experience or training did the researcher have? | All the interviewers were trained on the interview guideline and for analyzing the data. |
| Relationship with participants |  |  |  |
| 6. | Relationship established | Was a relationship established prior to study commencement? | the researchers did not meet the patients prior to the first approach for asking for participation. |
| 7. | Participant knowledge of the interviewer | What did the participants know about the researcher? e*.g. personal goals, reasons for doing the research* | What can be found on the institutional websites of the different affiliations.  We did not systematically inform the interviewees about our personal background and interests in the research, unless they asked about it. |
| 8. | Interviewer characteristics | What characteristics were reported about the interviewer/facilitator? e.g. *Bias, assumptions, reasons and interests in the research topic* | What can be found on the institutional websites of the different affiliations.  We did not systematically inform the interviewees about our personal background and interests in the research, unless they asked about it. |
| **Domain 2: study design** |  |  |  |
| Theoretical framework |  |  |  |
| 9. | Methodological orientation and Theory | What methodological orientation was stated to underpin the study? *e.g. grounded theory, discourse analysis, ethnography, phenomenology, content analysis* | The study was underpinned by a qualitative research methodology using semi-structures interviews complemented by quantitative data collected via questionnaires before the interviews.  We applied thematic analyses according to Braun and Clarke 2006 (see references).  See chapter ‘*Study design’* and ‘*Data analysis’* |
| Participant selection |  |  |  |
| 10. | Sampling | How were participants selected? *e.g. purposive, convenience, consecutive, snowball* | We performed a purposive sampling. |
| 11. | Method of approach | How were participants approached? e*.g. face-to-face, telephone, mail, email* | Face-to-face  See chapter ‘*study procedures’* |
| 12. | Sample size | How many participants were in the study? | 54, see chapter ‘*demographic and clinical characteristics’* and Table 1. |
| 13. | Non-participation | How many people refused to participate or dropped out? Reasons? | 4 dropouts due to worsened condition, lack of time, not reachable after informed consent. |
| Setting |  |  |  |
| 14. | Setting of data collection | Where was the data collected? e*.g. home, clinic, workplace* | Either at the hospitals (in-person) or via phone or video call. |
| 15. | Presence of non-participants | Was anyone else present besides the participants and researchers? | No |
| 16. | Description of sample | What are the important characteristics of the sample? *e.g. demographic data, date* | See Table 1. |
| Data collection |  |  |  |
| 17. | Interview guide | Were questions, prompts, guides provided by the authors? Was it pilot tested? | Yes, the interview guideline was pilot tested. |
| 18. | Repeat interviews | Were repeat interviews carried out? If yes, how many? | No. |
| 19. | Audio/visual recording | Did the research use audio or visual recording to collect the data? | The interviews were audio recorded. |
| 20. | Field notes | Were field notes made during and/or after the interview or focus group? | Yes, during the interview and reported within the interview form. |
| 21. | Duration | What was the duration of the interviews or focus group? | On average 30 minutes. See chapter ‘*study procedures’*. |
| 22. | Data saturation | Was data saturation discussed? | Yes, several times during the regular meetings among all study sites. |
| 23. | Transcripts returned | Were transcripts returned to participants for comment and/or correction? | No, but the results were discussed with stakeholders (healthcare professionals) |
| **Domain 3: analysis and findings**z |  |  |  |
| Data analysis |  |  |  |
| 24. | Number of data coders | How many data coders coded the data? | 3; see chapter *‘CRediT authorship contribution statement’* |
| 25. | Description of the coding tree | Did authors provide a description of the coding tree? | Yes, see figure 1 |
| 26. | Derivation of themes | Were themes identified in advance or derived from the data? | From data, see chapter ‘*Data analysis’* and ‘*Themes*’. |
| 27. | Software | What software, if applicable, was used to manage the data? | Qualitative data: Nvivo software (version 12, QRS International).  Quantitative data: SPPS version 27 |
| 28. | Participant checking | Did participants provide feedback on the findings? | The results from the interviews were discussed with stakeholders (healthcare professionals) |
| Reporting |  |  |  |
| 29. | Quotations presented | Were participant quotations presented to illustrate the themes / findings? Was each quotation identified? e*.g. participant number* | Yes, see chapter ‘*Results’* |
| 30. | Data and findings consistent | Was there consistency between the data presented and the findings? | Yes, see chapters ‘*Results*’ and ‘*Discussion’* |
| 31. | Clarity of major themes | Were major themes clearly presented in the findings? | Yes, see chapter ‘*Results*’. |
| 32. | Clarity of minor themes | Is there a description of diverse cases or discussion of minor themes? | Yes, see chapter ‘*Discussion*’. |

Allison Tong, Peter Sainsbury, Jonathan Craig, Consolidated criteria for reporting qualitative research (COREQ): a 32-item checklist for interviews and focus groups, International Journal for Quality in Health Care, Volume 19, Issue 6, December 2007, Pages 349–357, <https://doi.org/10.1093/intqhc/mzm042>
