# Supplementary material for: Cancer care during the Covid-19 pandemic from the perspective of patients and their relatives: A qualitative study
Source: Heliyon. 2023 Sep 14;9(9):e19752. doi: 10.1016/j.heliyon.2023.e19752 (PMC10559054; doi:10.1016/j.heliyon.2023.e19752)
Supplement: Multimedia component 2 [file mmc2.docx]

**Questionnaire before interview**

Name:

…………………………………………………………………………………………………………………………………………………………….

What is your age?

………………………………………………………………………………………………………………………………………………….…………

What is your relationship with your relative who is also participating in this study?

…………………………………………………………………………………………………………………………………………………………….

Work status

□ employed

□ retired

□ (temporary) incapacitated

□ unemployed

Marital status:

□ married

□ registrated partnership

□ unmarried

□ divorced

□ widow(er)

**Distress Thermometer**

Instructions: please circle the number (0-10) that best describes how much distress you have been experiencing in the past week including today.


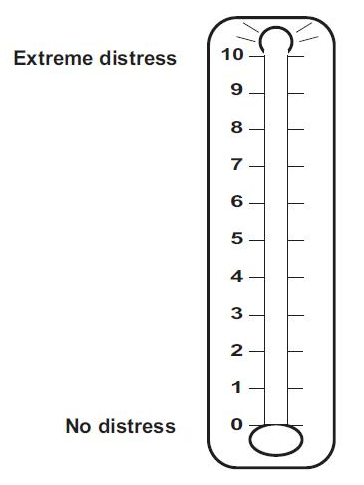


**CD RISC 2**

Instructions: For each question, check the box that best indicates how much you agree with the statement - it concerns the past month. If a situation has not happened recently, please answer how you think you would have felt.

|  | Not true at all | Seldom true | Some  times true | Often true | Almost allways true |
| --- | --- | --- | --- | --- | --- |
| 1. I am able to adapt when changes occur. |  |  |  |  |  |
| 2. I tend to bounce back after illness, injury, or otherwise difficult situations. |  |  |  |  |  |

**Questionnaire interview**

1. Can you tell us how you experienced the outbreak of the COVID-19 pandemic?

A. How has it affected your daily life?

2. Are you (were) afraid of becoming infected with COVID-19?

A. Can you tell me why or why not?

B. If so, how does/did the fear of infection affect you?

C. Has anyone in your immediate circle been infected?

3. Has anything changed in the care for your disease since the outbreak of the COVID-19 pandemic (care provided, appointments, treatment, follow-up, acute care/symptom management, continuity of care)?

A. If so, can you tell us about the changes you have experienced?

B. If not, would you like certain things to have changed and why?

4. How did you experience the appointments with the healthcare providers during the COVID-19 pandemic?

A. In what ways (physical, telephone, video, email) have you had contact with healthcare providers? How did you experience contact in this way(s)?

B. Would you have liked this way of contact to be different? If so, why?

5. How did you experience that your loved one(s) could not attend appointments or treatments?

6. What are/were your experiences with the communication from healthcare providers about the consequences of COVID-19 on your disease trajectory?

A. Are there things you would like to see changed in your communication with healthcare providers? If so, what would you have liked differently?

7. How would you describe the support you have received from healthcare providers during the COVID-19 pandemic?

A. Is there anything else the healthcare providers (could have) done to support/help you?

8. Are you concerned about your illness or care during the COVID-19 pandemic?

A. Who has helped you most to deal with these concerns?

9. Do you feel safe in Erasmus MC (during physical appointments, examinations or treatments) when it comes to COVID-19?

10. What are/were the most basic sources of information about your illness and the consequences of COVID-19?

A. Why is/was this resource useful to you?

B. Are there any issues you would have liked more or different information about (your illness/treatment, COVID-19 or care planning)?

11. During the COVID-19 pandemic, have you noticed a change (generally, in your illness or treatment setting) that you experienced as positive?

A. If so, can you explain in more detail?

12. How do you feel about vaccination?

A. What do you expect from the vaccination for your own situation?

B. Have you been vaccinated?

i. If 'no': Are you planning to get vaccinated? Why?

ii. If 'yes': What does the vaccination mean for you and your loved ones?

13. Do you have any ideas or suggestions on how to improve cancer-related care in a next wave or future pandemic?

14. Returning to the questionnaire you completed, summary of this interview: can you explain your answers (specify answer)?

A. The amount of physical/emotional/social/practical experience (score 0-10).

B. I am able to adapt when changes occur

C. I have the progression to walk again after illness, injury or other expiring situations.
